# Carcinoid crisis in Lutetium-177-Dotatate therapy of neuroendocrine tumors: an overview of pathophysiology, risk factors, recognition, and treatment

**DOI:** 10.1186/s41824-024-00216-6

**Published:** 2024-09-13

**Authors:** Stephen J. Sozio, William Raynor, Murray C. Becker, Anthony Yudd, Jeffrey S. Kempf

**Affiliations:** Department of Radiology, Rutgers Robert Wood Johnson Medical School, New Brunswick, NJ USA

**Keywords:** Lutetium-177-Dotatate, Carcinoid Crisis, Theragnostic, Radiotherapy

## Abstract

**Purpose:**

Lutetium-177-Dotatate (Lutathera^®^) is a combined radionuclide-peptide that is FDA-approved for the treatment of well-differentiated, somatostatin receptor-positive, gastroenteropancreatic neuroendocrine tumors. Carcinoid crisis is a rare, but potentially life-threatening risk of this radiopharmaceutical, of which prompt recognition and treatment is essential to reducing morbidity. This manuscript provides an overview of the topic to promote awareness of this adverse event, with emphasis on early recognition and management. In addition, we present our institution’s experience with Lutetium-177-Dotatate-associated complications across a five-year period.

**Methods:**

A literature review of lutetium-177-dotatate therapy and its potential implication of carcinoid crisis was performed. Additionally, a review of our institution’s experience is presented.

**Results:**

The incidence of carcinoid crisis induced by Lutetium-177-Dotatate therapy is estimated to range between 1 and 2% of treatment recipients. Those who have tumors located within the midgut, higher tumor burden, and the presence of metastasis have an increased risk of developing carcinoid crisis, among other risk factors. Carcinoid crisis is most often encountered within 12–48 h of receiving the first treatment dose, with the most common symptoms being nausea/vomiting, flushing, and diarrhea.

**Conclusion:**

Carcinoid crisis is a rare but potentially life-threatening complication of Lutetium-177-Dotatate therapy. Knowledge of risk factors and prompt recognition of symptoms is essential to successful treatment, with early initiation of intravenous octreotide serving a critical step in reducing morbidity of this adverse event.

**Supplementary Information:**

The online version contains supplementary material available at 10.1186/s41824-024-00216-6.

## Background

Neuroendocrine tumors (NET) are a diverse group of malignancies that account for approximately 1% of all malignancies diagnosed in the United States each year, the equivalent of approximately 12,000 patients (Dasari et al. 2017). Management of such tumors can be complex, involving a combination of surgical resection, chemotherapy, organ-targeted chemoembolization, and/or radionuclide therapy (Modlin et al. 2008).

Lutetium-177-Dotatate (Lutathera^®^) is a combined radionuclide-peptide that received FDA approval for the treatment of well-differentiated, somatostatin receptor-positive, gastroenteropancreatic NETs in 2018 (Love et al. 2022; Jia et al. 2022; Strosberg et al. 2018). While multiple studies have demonstrated efficacy of this radionuclide in the treatment of NETs, this therapy is not without risk, including a rare, but potentially life-threatening complication of carcinoid crisis (Jia et al. 2022; Kendi et al. 2019). Given its potential morbidity and mortality, carcinoid crisis remains imperative for clinicians to become educated and vigilant for its signs and symptoms so that treatment may be instituted rapidly. This manuscript provides an overview of the topic to promote awareness of this potential adverse event, with emphasis on early recognition and rapid management. In addition, we present our own institution’s experience Lutetium-177-Dotatate across a five year period.

## Methods

A literature review of PubMed^®^-indexed, peer-reviewed works centered on lutetium-177-dotatate therapy and its potential complication of carcinoid crisis was performed. Topics searched included, but were not limited to, incidence, pathophysiology, presentation, symptomatic management, and prevention of the adverse event, including relevant medication(s) and dosages. Additionally, a review of our institution’s experience was performed utilizing a radiology-specific clinical analytics application to search for adverse events in patients having undergone lutetium-177-dotatate therapy from October 2018 (the date of the first therapy our institution administered) to February 2024.

## Findings

### Overview of Neuroendocrine Tumors

Neuroendocrine tumors (NETs) are a diverse group of neoplasms which are unified by the commonality of being of neuroendocrine origin. NETs may cause symptoms such as the carcinoid syndrome by secreting biologically active peptides and amines that include serotonin, histamine, prostaglandins tachykinins, and kallikrein (Rogoza et al. 2022). NETs are most commonly located in the intestine, specifically the small intestine, in addition to the pancreas, lung, and adrenal glands, including tumors such as carcinoid and pheochromocytoma (Rindi and Wiedenmann 2012; Hofman et al. 2015; Costanzi et al. 2021; Halfdanarson et al. 2020).

Many NETs express somatostatin subtype type 2 receptors on their cell surface, which when bound to somatostatin, inhibits secretion of hormones through inhibition of intracellular cAMP and calcium ion release, and inhibits cell proliferation by upregulating the cell cycle inhibitors p27 and p21 (Rogoza et al. 2022). Tumors expressing somatostatin receptors can be identified in vivo by using nuclear medicine imaging, specifically using radiotracers which bind to somatostatin receptors, such as Gallium-68-Dotatate in PET imaging, and historically with Indium-111-Pentetreotide in traditional gamma camera imaging (Kendi et al. 2019). Given the ability to control both tumor cell division and hormone secretion through the somatostatin receptor, the development of somatostatin analogs has become a key target in non-surgical therapy and is the fundamental principal behind Lutetium-177-Dotatate therapy (Love et al. 2022).

Treatment plans for neuroendocrine tumors typically begins with the surgical pathology assessment of tumor aggressiveness that is based on assessing mitotic count, intracellular Ki-67 protein levels, and morphologic features of differentiation, including higher mitotic count, higher Ki-67 levels, and poorly differentiated histological morphology being associated with more aggressive tumor and poorer prognosis (Rogoza et al. 2022). In addition to tumor histological and molecular characterization, the choice of treatment modalities is also dependent on the anatomic location(s) of tumor and extent of disease (Rindi and Wiedenmann 2012). Surgical resection is typically reserved for non-metastatic NETs, while those suffering metastasis are typically treated with a combination of chemotherapy, organ-targeted chemoembolization, and/or radionuclide therapy (Rindi and Wiedenmann 2012).

### Fundamentals of Lutetium-177-Dotatate

Lutetium-177-Dotatate (Lutathera^®^) is a combined radionuclide-peptide, which selectively binds to and is internalized by cells expressing somatostatin subtype 2 (SSR2) receptor. The radionuclide’s beta-emission results in free radical generation and subsequent DNA destruction, leading to cell death. Lutetium-177-Dotatate has been shown to lead to longer progression-free and overall survival, as evidenced in the NETTER-1 trial (Love et al. 2022; Jia et al. 2022; Strosberg et al. 2018).

Lutetium-177-Dotatate therapy requires a pretreatment diagnostic Gallium-68-Dotatate PET/CT imaging study to demonstrate that the tumor is Dotatate-avid (Hofman et al. 2015). The standard FDA-approved treatment protocol of Lutetium-177-Dotatate is 7.4 GBq (200 mCi) administered intravenously in 4 separate occasions across 8-week intervals. To promote radiopharmaceutical uptake by the tumor, long-acting somatostatin is discontinued for 4 weeks prior to therapy, and short acting somatostatin is discontinued 24 h prior to therapy (Jia et al. 2022; Strosberg et al. 2018).

While multiple studies have demonstrated efficacy of Lutetium-177-Dotatate in the treatment of NETs, this therapy is not without risk, with the most common adverse effects being nausea, vomiting, and abdominal pain, and with rarer complications including renal impairment and myelodysplastic syndrome (Love et al. 2022; Jia et al. 2022; Strosberg et al. 2018; Kendi et al. 2019; Keizer et al. 2008). Of importance, carcinoid crisis is a rare but potentially life-threatening complication of therapy.

### Lutetium-177-Dotatate-induced Carcinoid Crisis

Lutetium-177-Dotatate induced carcinoid crisis arises when radiotherapy induction results in a sudden massive release of over 40 hormones from tumor cells, most notably serotonin, histamine, and catecholamines (Gade et al. 2020). Tumor lysis is the presumed etiology of carcinoid crisis after radionuclide therapy. The incidence of carcinoid crisis is estimated to range between 1 and 2% of treatment recipients, although the exact incidence is not well known, in part due to poor definition of criteria qualifying the diagnosis of this adverse event (Kendi et al. 2019; Keizer et al. 2008; Tapia Rico et al. 2018). Those suffering tumors located within the small intestine or right-sided colon, higher tumor burden, metastatic disease to the liver, and high levels of serum or urine 5-hydroxyindolacetic acid and serum chromogranin A were found to be at higher risk of developing carcinoid crisis, as illustrated in Table 1 (Jia et al. 2022; Rogoza et al. 2022; Keizer et al. 2008; Gade et al. 2020; Tapia Rico et al. 2018; Olmo-Garcia et al. 2020).

Carcinoid crisis is most often encountered within 12–48 h of receiving the first treatment dose, however it has also been observed in patients after receiving subsequent treatment doses (Tapia Rico et al. 2018; olmo-Garcia et al. 2020). A summary of the most common symptoms of carcinoid crisis are summarized in table 2, with the most common symptoms encountered being nausea/vomiting, severe diarrhea, and flushing (Gade et al. 2020). Of note, there have been no documented deaths attributable to Lu-177-dotatate-induced carcinoid crisis as of May 2024 (olmo-Garcia et al. 2020).

**Table 1:** Risk factors for developing Lutetium-177-dotatate-induced Carcinoid Crisis

Tumors within the midgut (small intestine, right-sided colon)
Higher tumor burden
Liver metastases
High levels of 5-hydroxyindolacetic acid and chromogranin A
History of carcinoid syndrome, including carcinoid heart disease
Advanced age
Concurrent use of drugs which promote histamine release (β2 agonists)

**Table 2:** Symptoms of Lutetium-177-dotatate-induced Carcinoid Crisis

Nausea/vomiting (most common)
Severe diarrhea
Flushing
Tachycardia
Altered Mental status
Transaminitis
Anemia
Electrolyte Disturbance

### Management of Lutetium-177-dotatate-induced Carcinoid Crisis

Management of Lutetium-177-Dotatate-induced Carcinoid Crisis follows the same approach to treatment as carcinoid crisis caused by other etiologies. Management begins with enhanced alertness for symptoms of carcinoid crisis, as early recognition and intervention has been associated with more favorable outcomes. Pre-treatment review of risk factors, as illustrated in Table 1, should prompt enhanced vigilance in those with a greater predisposition for developing this adverse event.

In addition to maintaining airway and circulatory system patency, the single most important step in management of carcinoid crisis is the prompt administration of intravenous octreotide, a somatostatin analog which directly inhibits the release of vasoactive amines from tumor cells, while simultaneously acting as a somatostatin analog to inhibit splanchnic blood flow and the release of other hormones, such as insulin and glucagon (Dhanani et al. 2020; Baradasi et al. 2022). Octreotide is administered at an intravenous bolus of 500–1000 µg at the onset of symptoms, and repeated at 5-minute intervals until control of symptoms is achieved (Tapia Rico et al. 2018). If symptoms remain refractory despite multiple bolus doses of octreotide, continuous intravenous infusion can be initiated and titrated until symptom improvement (Tapia Rico et al. 2018). Review of literature revealed no consensus on the number of bolus doses which should be attempted/administered prior to escalating to a continuous octreotide infusion (Tapia Rico et al. 2018; Olmo-Garcia et al. 2020; Mittra 2018). In addition to octreotide, serotonin modulators may be initiated as either second-line or combination first-line therapy to directly block hormone effect, including agents such as cyproheptadine, a 5-HT2A receptor inhibitor, and telotristat ethyl, a tryptophan hydroxylase inhibitor which hinders the conversion of tryptophan to serotonin (Hofman et al. 2015; Tapia Rico et al. 2018; Olmo-Garcia et al. 2020; Mittra 2018). Additional symptomatic control should be considered, including anti-diarrheal agents, H1/H2 blockers, and/or anxiolytics (Tapia Rico et al. 2018; Olmo-Garcia et al. 2020; Mittra 2018).

### Prevention of Lutetium-177-dotatate-induced Carcinoid Crisis

A number of preemptive measures have been documented in the effort to prospectively prevent development of Lu-177-Dotatate-induced carcinoid crisis. One of the most well-documented approaches involves the pre-treatment administration of octreotide, which has demonstrated efficacy in reducing incidence of carcinoid crisis (Olmo-Garcia et al. 2020). Patients presenting with a higher number of risk factors for carcinoid crisis prior to undergoing treatment may be prophylactically initiated on octreotide long-acting release (LAR) at a dose of 10–30 mg, delivered intramuscularly and administered every 28 days (Olmo-Garcia et al. 2020; Cheng et al. 2023). In contrast, those who do not experience pre-treatment carcinoid syndrome/crisis, but are at increased risk for developing Lu-177-Dotatate-induced carcinoid crisis, may receive a subcutaneous bolus of octreotide (non-LAR formulation) at a dose of 250–500 µg, or an intravenous dose of 50 µg, to be administered within 1–2 h before the procedure (Tapia Rico et al. 2018; Olmo-Garcia et al. 2020; Baradasi et al. 2022; Burkett et al. 2020). It is important to note that octreotide and octreotide-LAR pre-treatment is not without risk, as several sources have documented that administration of octreotide or octreotide-LAR within 48 h or 28 days prior to Lu-177-Dotate (respectively) may interfere with therapy efficacy via direct competitive inhibition for uptake into NET cells (Tapia Rico et al. 2018; Olmo-Garcia et al. 2020; Hicks et al. 2017). The decision to initiate pre-treatment octreotide is centered around risk/benefit analysis for each patient, and should be strongly considered when benefits outweigh risks, specifically in those with high risk of developing carcinoid crisis (Tapia Rico et al. 2018; Rolleman et al. 2007). Additional pharmacologic pre-treatment may be achieved with dexamethasone and/or selective 5-hydroxytryptamine 3 receptor antagonists, to reduce both systemic inflammatory response and response to vasoactive amines which precipitate carcinoid crisis (Burkett et al. 2020).

Prevention of carcinoid crisis can also be achieved through pre-treatment tumor debulking, either by surgery, interventional radiology, or external-beam radiation therapy, thereby reducing tumor burden and the number amine-producing tumor cells (Olmo-Garcia et al. 2020). Correction of nutritional deficiencies, electrolyte disturbances, and hypoproteinemia prior therapy has demonstrated efficacy in prevention of carcinoid crisis, although their proposed mechanisms are not unique or specific to the pathogenesis of carcinoid crisis (Tapia Rico et al. 2018; Olmo-Garcia et al. 2020; Burkett et al. 2020). Furthermore, avoidance of exercise, and co-administration of an amino acid infusion rich in lysine and arginine have also been documented to lessen incidence of carcinoid crisis (Tapia Rico et al. 2018; Olmo-Garcia et al. 2020). Pretreatment steroid therapy has been proposed as a method to reduce carcinoid crisis in patients at highest risk (Table 1), but has yet to become standard of care (Tapia Rico et al. 2018).

For patients who have experienced a prior complication of Lu-177-Dotatate-induced carcinoid crisis, there is no clear consensus within the literature whether therapy should be re-attempted with or without pretreatment, or whether patients are at increased risk for subsequence episodes of carcinoid crisis.

### Institutional experience in Lutetium-177-dotatate-induced Carcinoid Crisis

Review of our own institutional history of administering Lutetium-177-Dotatate therapy revealed a total of 127 administrations of therapy between October 2018 to February 2024, with no reported complications of carcinoid crisis. One adverse event was noted in a 64-year-old female suffering metastatic endobronchial carcinoid of the trachea and bilateral central bronchi, who developed moderate dyspnea and acute hypoxic respiratory failure approximately 2 h after receiving her fourth administration of Lutetium-177-Dotatate. She did not experience any adverse events following prior administrations of Lutetium-177-Dotatate, but had reported worsening exertional dyspnea over several months leading up to her first dose of Lutetium-177-Dotatate. She did not experience flushing, diarrhea, or hypotension following any incidence of Lutetium-177-Dotatate therapy. She was admitted to the hospital under observation for 1 day and treated with supplemental oxygen therapy, with subsequent improvement of her symptoms and return to her baseline within 24 h without additional intervention. Her symptoms were ultimately attributed to bronchospasm, possibly with a component of tracheal/bronchial obstruction given the location of her tumor. Carcinoid crisis was not suspected, given the lack of adverse event occurrence upon receiving the first three doses of Lutetium-177-Dotatate, combined with lack of flushing, diarrhea, or hypotension.

## Conclusion

Carcinoid crisis is a rare but potentially life-threatening complication of Lutetium-177-Dotatate therapy, most commonly presenting as acute onset nausea/vomiting, diarrhea, flushing, and/or tachycardia. Neuroendocrine tumors of the midgut, high tumor burden, and presence of liver metastases are among several risk factors for developing carcinoid crisis. Knowledge of risk factors and prompt recognition of symptoms is essential to successful treatment, with early initiation of intravenous octreotide serving a critical step in reducing morbidity of this adverse event.

A summary of salient references included in this review and their corresponding core relevance(s) in this review, are presented in Table 3.

**Table 3:** Summary of salient references and corresponding core relevance(s)

Reference	Relevance
Jia AY, Kashani R, Zaorsky NG, et al. “Lutetium-177 DOTATATE: A Practical Review.” *Practl Radiat Oncol*, 2022; 12(4):305 − 11.	Background
Kendi A, Tuba TR, Halfdanarson AP, et al. “Therapy With 177Lu-DOTATATE: Clinical Implementation and Impact on Care of Patients With Neuroendocrine Tumors.” *Am J Roentg*, 2019; 213(2):309 − 17.	Background, Symptomatology, Management, Prevention
de Keizer B, van Aken MO, Feelders RA, et al. “Hormonal crises following receptor radionuclide therapy with the radiolabeled somatostatin analogue [^177^Lu-DOTA0,Tyr3]octreotate.” *Eur J Nucl Med Mol Imaging*, 2008; 35:749–755.	Background, Risk Factors, Symptomatology, Management, Prevention
Gade AK, Olariu E, Douthit NT. “Carcinoid Syndrome: A Review.” *Cureus*, 2020; 12(3):e7186.	Symptomatology, Management, Prevention
Tapia Rico G, Li M, Pavlakis N, Cehic G, Price TJ. “Prevention and management of carcinoid crises in patients with high-risk neuroendocrine tumours undergoing peptide receptor radionuclide therapy (PRRT): literature review and case series from two Australian tertiary medical institutions.” *Cancer Treat Rev*, 2018; 66:1–6.	Background, Risk Factors, Symptomatology, Management, Prevention
del Olmo-Garcia MI, Muros MA, Lopez-de-la-Torre M, et al. “Prevention and Management of Hormonal Crisis during Theragnosis with LU-DOTA-TATE in Neuroendocrine Tumors. A Systematic Review and Approach Proposal.” *J Clin Med*, 2020; 9(7).	Background, Risk Factors, Symptomatology, Management, Prevention
Dhanani J, Pattison DA, Burge M, et al. “Octreotide for resuscitation of cardiac arrest due to carcinoid crisis precipitated by novel peptide receptor radionuclide therapy (PRRT): A case report.” *J Crit Care*, 2020; 60:318–322.	Management
Baradasi C, Benatti S, Luppi G, Garajova I, Piacentini F, Dominici M, Gelsomino F. “Carcinoid Crisis: A Misunderstood and Unrecognized Oncological Emergency.” *Cancers*, 2022; 14(3):662.	Management
Mittra E. “Neuroendocrine Tumor Therapy: 177Lu-DOTATATE.” *AJR Am J Roentgenol*, 2018; 211(2):278 − 85.	Symptomatology, Management, Prevention
Cheng Y, Anthony L, Delcher C, Moga DC, Chauhan A, Huang B, Adams V. “Prescribing Characteristics of Octreotide Immediate-Release and Long-Acting Release in Patients with Neuroendocrine Tumors.” *Oncologist*, 2023; 28(6):479 − 85.	Prevention
Burkett BJ, Dundar A, Young JR, et al. “How We Do It: A Multidisciplinary Approach to 177Lu DOTATATE Peptide Receptor Radionuclide Therapy.” *Radiol*, 2020; 298(2):261–274.	Prevention

## Electronic supplementary material

Below is the link to the electronic supplementary material.

Supplementary Material 1

Supplementary Material 2

## Data Availability

Data sharing not applicable to this article as no datasets were generated or analyzed during the current study.
