# Supplementary material for: Carcinoid crisis in Lutetium-177-Dotatate therapy of neuroendocrine tumors: an overview of pathophysiology, risk factors, recognition, and treatment
Source: EJNMMI Rep. 2024 Sep 13;8(1):29. doi: 10.1186/s41824-024-00216-6 (PMC11393224; doi:10.1186/s41824-024-00216-6)
Supplement: Supplementary file 1 — Supplementary Material 1 [file 41824_2024_216_MOESM1_ESM.docx]

Carcinoid Crisis in Lutetium-177-Dotatate Therapy of Neuroendocrine Tumors:

An Overview of Pathophysiology, Risk Factors, Recognition, and Treatment

Stephen J. Sozio, DO, MBS^1^, William Raynor, MD^1^, Murray C. Becker, MD, PhD^1^, Anthony Yudd, MD, PhD^1^,

Jeffrey S. Kempf, MD, FACR^1^

1. Rutgers Robert Wood Johnson Medical School, New Brunswick, NJ USA

Corresponding Author:

Stephen J. Sozio, DO, MBS

Rutgers Robert Wood Johnson Medical School

1 Robert Wood Johnson Place

Department of Radiology

New Brunswick, NJ 08901 USA

E-Mail: [sjs335@rwjms.rutgers.edu](mailto:sjs335@rwjms.rutgers.edu)

Phone: (732) 235-7721

Acknowledgements: None

Keywords: Lutetium-177-Dotatate, Carcinoid Crisis, Theragnostic, Radiotherapy

Abstract

*Purpose*: Lutetium-177-Dotatate (Lutathera®) is a combined radionuclide-peptide that is FDA-approved for the treatment of well-differentiated, somatostatin receptor-positive, gastroenteropancreatic neuroendocrine tumors. Carcinoid crisis is a rare, but potentially life-threatening risk of this radiopharmaceutical, of which prompt recognition and treatment is essential to reducing morbidity. This manuscript provides an overview of the topic to promote awareness of this adverse event, with emphasis on early recognition and management. In addition, we present our institution’s experience with Lutetium-177-Dotatate-associated complications across a five-year period.

*Methods*: A literature review of lutetium-177-dotatate therapy and its potential implication of carcinoid crisis was performed. Additionally, a review of our institution’s experience is presented.

*Results*: The incidence of carcinoid crisis induced by Lutetium-177-Dotatate therapy is estimated to range between 1-2% of treatment recipients. Those who have tumors located within the midgut, higher tumor burden, and the presence of metastasis have an increased risk of developing carcinoid crisis, among other risk factors. Carcinoid crisis is most often encountered within 12-48 hours of receiving the first treatment dose, with the most common symptoms being nausea/vomiting, flushing, and diarrhea.

*Conclusion*: Carcinoid crisis is a rare but potentially life-threatening complication of Lutetium-177-Dotatate therapy. Knowledge of risk factors and prompt recognition of symptoms is essential to successful treatment, with early initiation of intravenous octreotide serving a critical step in reducing morbidity of this adverse event.

Background

Neuroendocrine tumors (NET) are a diverse group of malignancies that account for approximately 1% of all malignancies diagnosed in the United States each year, the equivalent of approximately 12,000 patients [1]. Management of such tumors can be complex, involving a combination of surgical resection, chemotherapy, organ-targeted chemoembolization, and/or radionuclide therapy [2].

Lutetium-177-Dotatate (Lutathera®) is a combined radionuclide-peptide that received FDA approval for the treatment of well-differentiated, somatostatin receptor-positive, gastroenteropancreatic NETs in 2018 [3,4,5]. While multiple studies have demonstrated efficacy of this radionuclide in the treatment of NETs, this therapy is not without risk, including a rare, but potentially life-threatening complication of carcinoid crisis [4,6]. Given its potential morbidity and mortality, carcinoid crisis remains imperative for clinicians to become educated and vigilant for the signs and symptoms of carcinoid crisis so that treatment may be instituted rapidly. This manuscript provides an overview of the topic to promote awareness of this potential adverse event, with emphasis on early recognition and rapid management. In addition, we present our own institution’s experience Lutetium-177-Dotatate across a five year period.

Methods

A literature review of PubMed®-indexed, peer-reviewed works centered on lutetium-177-dotatate therapy and its potential complication of carcinoid crisis was performed. Topics searched included, but were not limited to, incidence, pathophysiology, presentation, symptomatic management, and prevention of the adverse event, including relevant medication(s) and dosages,. Additionally, a review of our institution’s experience was performed utilizing a radiology-specific clinical analytics application search of adverse events in patients having undergone lutetium-177-dotatate therapy from October 2018 (the date of the first therapy our institution administered) to February 2024.

Findings

*Overview of Neuroendocrine Tumors*

Neuroendocrine tumors (NETs) are a diverse group of neoplasms which are unified by the commonality of being of neuroendocrine origin. NETs may cause symptoms such as the carcinoid syndrome by secreting biologically active peptides and amines that include serotonin, histamine, prostaglandins tachykinins, and kallikrein [7]. NETs are most commonly located in the intestine, specifically the small intestine, in addition to the pancreas, lung, and adrenal glands, including tumors such as carcinoid and pheochromocytoma [8,9,10,11].

Many NETs express subtype somatostatin type 2 receptors on their cell surface, which when bound to somatostatin, inhibits secretion of hormones through inhibition of intracellular cAMP and calcium ion release, and inhibits cell proliferation by upregulating the cell cycle inhibitors p27 and p21 [7]. Tumors expressing somatostatin receptors can be identified in vivo by using nuclear medicine imaging, specifically using radiotracers which bind to somatostatin receptors, such as Gallium-68-Dotatate in PET imaging, and previously with Indium-111-Pentetreotide in traditional gamma camera imaging [6]. Given the ability to control both tumor cell division and hormone secretion through the somatostatin receptor, the development of somatostatin analogs has become a key target in non-surgical therapy and is the fundamental principal behind Lutetium-177-Dotatate therapy [3].

Treatment plans for neuroendocrine tumors typically begins with the surgical pathology assessment of tumor aggressiveness that is based on assessing mitotic count, intracellular Ki-67 protein levels, and morphologic features of differentiation, e.g., higher mitotic count, higher Ki-67 levels, and poorly differentiated histological morphology being associated with more aggressive tumor and poorer prognosis [7]. In addition to tumor histological and molecular characterization, the choice of treatment modalities is also dependent on the anatomic location(s) of tumor and extent of disease [8]. Surgical resection is typically reserved for non-metastatic NETs, while those suffering metastasis are typically treated with a combination of medical therapy, organ-targeted chemoembolization, and/or radionuclide therapy [8].

*Fundamentals of Lutetium-177-Dotatate*

Lutetium-177-Dotatate (Lutathera®) is a combined radionuclide-peptide, which selectively binds to and is internalized by cells expressing somatostatin (SSR2) receptor. The radionuclide’s subsequent beta-emission results in free radical generation and subsequent DNA destruction leading to cell death. Lutetium-177-Dotatate has been shown to lead to longer progression-free and overall survival, as evidenced in the NETTER-1 trial [3,4,5].

Lutetium-177-Dotatate therapy requires a pretreatment diagnostic Gallium-68-Dotatate PET/CT imaging study to demonstrate that the tumor is Dotatate-avid [9]. The standard FDA-approved treatment protocol of Lutetium-177-Dotatate is 7.4 GBq (200 mCi) administered intravenously in 4 separate occasions across 8-week intervals. To promote radiopharmaceutical uptake by the tumor, long-acting somatostatin is discontinued for 4 weeks prior to therapy, and short acting somatostatin is discontinued 24 hours prior to therapy [4,5].

While multiple studies have demonstrated efficacy of Lutetium-177-Dotatate in the treatment of NETs, this therapy is not without risk, with the most common adverse effects being nausea, vomiting, and abdominal pain, and with rarer complications including renal impairment and myelodysplastic syndrome [3,4,5,6,12]. Of importance, carcinoid crisis is a rare but potentially life-threatening complication of therapy.

*Lutetium-177-Dotatate-induced Carcinoid Crisis*

Lutetium-177-Dotatate induced carcinoid crisis arises when radiotherapy induction results in a sudden massive release of over 40 hormones, most notably serotonin, histamine, and catecholamines, from tumor cells [13]. Tumor lysis is the presumed etiology of carcinoid crisis after radionuclide therapy. The incidence of carcinoid crisis is estimated to range between 1-2% of treatment recipients, although the exact incidence is not well known, in part due to poor definition of criteria qualifying diagnosis of this adverse event [6,12,14]. Those suffering tumors located within the small intestine or right-sided colon, higher tumor burden, metastatic disease to the liver, and high levels of serum or urine 5-hydroxyindolacetic acid and serum chromogranin A were found to be at higher risk of developing carcinoid crisis, as illustrated in Table 1 [4,7,12,13,14,15].

| Tumors within the midgut (small intestine, right-sided colon) |
| --- |
| Higher tumor burden |
| Liver metastases |
| High levels of 5-hydroxyindolacetic acid and chromogranin A |
| History of carcinoid syndrome, including carcinoid heart disease |
| Advanced age |
| Concurrent use of drugs which promote histamine release (β2 agonists) |

Table 1: Risk Factors for Developing Lutetium-177-Dotatate-induced Carcinoid Crisis.

Carcinoid crisis is most often encountered within 12-48 hours of receiving the first treatment dose, however it also has been observed in patients after receiving subsequent treatment doses [14,15]. A summary of the most common symptoms of carcinoid crisis are summarized in Table 2, with the most common symptoms encountered being nausea/vomiting, severe diarrhea, and flushing [13]. Of note, there have been no documented deaths attributable to Lu-177-Dotatate-induced carcinoid crisis as of May 2024 [15].

| Nausea/vomiting (most common) |
| --- |
| Severe diarrhea |
| Flushing |
| Tachycardia |
| Altered Mental status |
| Transaminitis |
| Anemia |
| Electrolyte Disturbance |

Table 2: Symptoms of Lutetium-177-Dotatate-induced Carcinoid Crisis.

*Management of* *Lutetium-177-Dotatate-induced Carcinoid Crisis*

Management of Lutetium-177-Dotatate-induced Carcinoid Crisis follows the same approach to treatment as carcinoid crisis caused by other etiologies. Management begins with enhanced alertness for symptoms of carcinoid crisis, as early recognition and intervention has been associated with more favorable outcomes. Pre-treatment review of risk factors, as illustrated in Table 1, should prompt enhanced vigilance in those with a greater predisposition for developing this adverse event.

In addition to maintaining airway and circulatory system patency, the single most important step in management of carcinoid crisis is the prompt administration of intravenous octreotide, a somatostatin analog which directly inhibits the release of vasoactive amines from tumor cells, while simultaneously acting as a somatostatin analog to inhibit splanchnic blood flow and the release of other hormones, such as insulin and glucagon [16,17]. Octreotide is administered at an intravenous bolus of 500–1000 μg at the onset of symptoms, and repeated at 5-minute intervals until control of symptoms is achieved [14]. If symptoms remain refractory despite multiple bolus doses of octreotide, continuous intravenous infusion can be initiated and titrated until symptom improvement [14]. Review of literature revealed no consensus on the number of bolus doses which should be attempted/administered prior to escalating to a continuous octreotide infusion [14,15,18]. In addition to octreotide, serotonin modulators may be initiated as either second-line or combination first-line therapy to directly block hormone effect, including agents such as cyproheptadine, a 5-HT2A receptor inhibitor, and telotristat ethyl, a tryptophan hydroxylase inhibitor which hinders the conversion of tryptophan to serotonin [9,14,15,18]. Additional symptomatic control should be considered, including anti-diarrheal agents, H1/H2 blockers, and/or anxiolytics [14,15,18].

*Prevention of* *Lutetium-177-Dotatate-induced Carcinoid Crisis*

A number of pre-emptive measures have been documented in the effort to prospectively prevent development of Lu-177-Dotatate-induced carcinoid crisis. One of the most well-documented approaches involves the pre-treatment administration of octreotide, which has demonstrated efficacy in reducing incidence of carcinoid crisis [15]. Patients presenting with a higher number of risk factors for carcinoid crisis prior to undergoing treatment may be prophylactically initiated on octreotide long-acting release (LAR) 10–30 mg intramuscularly administered every 28 days [15,19]. In contrast, those who do not experience pre-treatment carcinoid syndrome/crisis, but are at increased risk for developing Lu-177-Dotatate-induced carcinoid crisis, may receive a subcutaneous bolus of octreotide (non-LAR formulation) at a dose of 250–500 μg, or an intravenous dose of 50 μg, to be administered within 1–2 hours before the procedure [14,15,17,20]. While some sources have cited use of intravenous It is important to note that octreotide and octreotide-LAR pre-treatment is not without risk, as several sources have documented that administration of octreotide or octreotide-LAR within 48 hours or 28 days prior to Lu-177-Dotate (respectively) may interfere with therapy efficacy via direct competitive inhibition for uptake into NET cells [14,15,21]. The decision to initiate pre-treatment octreotide is centered around risk/benefit analysis, and should be strongly considered which benefits outweigh risks, specifically in those with high risk of developing carcinoid crisis [14,22]. Additional pharmacologic pre-treatment may be achieved with dexamethasone and/or selective 5-hydroxytryptamine 3 receptor antagonists, to reduce both systemic inflammatory response and response to vasoactive amines which precipitate carcinoid crisis [20].

Prevention of carcinoid crisis can also be achieved through pre-treatment tumor debulking, either by surgery, interventional radiology, or external-beam radiation therapy, thereby reducing tumor burden and the number amine-producing tumor cells [15]. Correction of nutritional deficiencies, electrolyte disturbances, and hypoproteinemia prior therapy has demonstrated efficacy in prevention of carcinoid crisis, although their proposed mechanisms are not unique or specific to the pathogenesis of carcinoid crisis [14,15,20]. Furthermore, avoidance of exercise, and co-administration of an amino acid infusion rich in lysine and arginine have also been documented to lessen incidence of carcinoid crisis [14,15]. Pretreatment steroid therapy has been proposed as a method to reduce carcinoid crisis in patients at highest risk (table 1), but has yet to become standard of care [14].

For patients who have experienced a prior complication of Lu-177-Dotatate-induced carcinoid crisis, there is no clear consensus within the literature whether therapy should be re-attempted with or without pretreatment, or whether patients are at increased risk for subsequence episodes of carcinoid crisis.

*Institutional Experience in Lutetium-177-Dotatate-induced Carcinoid Crisis*

Review of our own institutional history of administering Lutetium-177-Dotatate therapy revealed a total of 127 administrations of therapy between October 2018 to February 2024, with no reported complications of carcinoid crisis. One adverse event was noted in a 64-year-old female suffering metastatic endobronchial carcinoid of the trachea and bilateral central bronchi, who developed moderate dyspnea and acute hypoxic respiratory failure approximately 2 hours after receiving her fourth administration of Lutetium-177-Dotatate. She did not experience any adverse events following prior administrations of Lutetium-177-Dotatate, but had reported worsening exertional dyspnea over several months leading up to her first dose of Lutetium-177-Dotatate. She did not experience flushing, diarrhea, or hypotension following any incidence of Lutetium-177-Dotatate therapy. She was admitted to the hospital under observation for 1 day and treated with supplemental oxygen therapy, with subsequent improvement of her symptoms and return to her baseline within 24 hours without additional intervention. Her symptoms were ultimately attributed to bronchospasm, possibly with a component of tracheal/bronchial obstruction given the location of her tumor. Carcinoid crisis was not suspected, given the lack of adverse event occurrence upon receiving the first three doses of Lutetium-177-Dotatate, combined with lack of flushing, diarrhea, or hypotension.

Conclusion

Carcinoid crisis is a rare but potentially life-threatening complication of Lutetium-177-Dotatate therapy, most commonly presenting as acute onset nausea/vomiting, diarrhea, flushing, and/or tachycardia. Neuroendocrine tumors of the midgut, high tumor burden, and presence of liver metastases are among several risk factors for developing carcinoid crisis. Knowledge of risk factors and prompt recognition of symptoms is essential to successful treatment, with early initiation of intravenous octreotide serving a critical step in reducing morbidity of this adverse event.

A summary of salient references included in this review and their corresponding core relevance(s) in this review, are presented in Table 3.

| **Reference** | **Relevance** |
| --- | --- |
| Jia AY, Kashani R, Zaorsky NG, et al. “Lutetium-177 DOTATATE: A Practical Review.” *Practl Radiat Oncol*, 2022; 12(4):305-11. | Background |
| Kendi A, Tuba TR, Halfdanarson AP, et al. “Therapy With 177Lu-DOTATATE: Clinical Implementation and Impact on Care of Patients With Neuroendocrine Tumors.” *Am J Roentg*, 2019; 213(2):309-17. | Background, Symptomatology, Management, Prevention |
| de Keizer B, van Aken MO, Feelders RA, et al. “Hormonal crises following receptor radionuclide therapy with the radiolabeled somatostatin analogue [^177^Lu-DOTA0,Tyr3]octreotate.” *Eur J Nucl Med Mol Imaging,* 2008; 35:749–755. | Background, Risk Factors, Symptomatology, Management, Prevention |
| Gade AK, Olariu E, Douthit NT. “Carcinoid Syndrome: A Review.” *Cureus*, 2020; 12(3):e7186. | Symptomatology, Management, Prevention |
| Tapia Rico G, Li M, Pavlakis N, Cehic G, Price TJ. “Prevention and management of carcinoid crises in patients with high-risk neuroendocrine tumours undergoing peptide receptor radionuclide therapy (PRRT): literature review and case series from two Australian tertiary medical institutions.” *Cancer Treat Rev*, 2018; 66:1–6. | Background, Risk Factors, Symptomatology, Management, Prevention |
| del Olmo-Garcia MI, Muros MA, Lopez-de-la-Torre M, et al. “Prevention and Management of Hormonal Crisis during Theragnosis with LU-DOTA-TATE in Neuroendocrine Tumors. A Systematic Review and Approach Proposal.” *J Clin Med*, 2020; 9(7). | Background, Risk Factors, Symptomatology, Management, Prevention |
| Dhanani J, Pattison DA, Burge M, et al. “Octreotide for resuscitation of cardiac arrest due to carcinoid crisis precipitated by novel peptide receptor radionuclide therapy (PRRT): A case report.” *J Crit Care*, 2020; 60:318-322. | Management |
| Baradasi C, Benatti S, Luppi G, Garajova I, Piacentini F, Dominici M, Gelsomino F. “Carcinoid Crisis: A Misunderstood and Unrecognized Oncological Emergency.” *Cancers*, 2022; 14(3):662. | Management |
| Mittra E. “Neuroendocrine Tumor Therapy: 177Lu-DOTATATE.” *AJR Am J Roentgenol*, 2018; 211(2):278-85. | Symptomatology, Management, Prevention |
| Cheng Y, Anthony L, Delcher C, Moga DC, Chauhan A, Huang B, Adams V. “Prescribing Characteristics of Octreotide Immediate-Release and Long-Acting Release in Patients with Neuroendocrine Tumors.” *Oncologist*, 2023; 28(6):479-85. | Prevention |
| Burkett BJ, Dundar A, Young JR, et al. “How We Do It: A Multidisciplinary Approach to 177Lu DOTATATE Peptide Receptor Radionuclide Therapy.” *Radiol*, 2020; 298(2):261-274. | Prevention |

Table 3: Summary of salient references and corresponding core relevance(s).

References

1. Dasari A, Shen C, Halperin D, et al. “Trends in the Incidence, Prevalence, and Survival Outcomes in Patients with Neuroendocrine Tumors in the United States.” *JAMA Oncol*, 2017;3(10):1335–1342.
2. Modlin IM, Kjell Oberg DC, Jensen R, et al. “Gastroenterohepatic Neuroendocrine Tumors.” *Lancet Oncol*, 2008; 9(1):61-72.
3. Love C, Desai NB, Abraham T, et al. “ACR-ACNM-ASTRO-SNMMI Practice Parameter for Lutetium-177 (Lu-177) DOTATATE Therapy.” *Am J Clin Oncol,* 2022; 45(6):233-42.
4. Jia AY, Kashani R, Zaorsky NG, et al. “Lutetium-177 DOTATATE: A Practical Review.” *Practl Radiat Oncol*, 2022; 12(4):305-11.
5. Strosberg J, El-Haddad G, Wolin E, et al. “Phase 3 Trial of 177Lu-Dotatate for Midgut Neuroendocrine Tumors.” *N Eng J Med*, 2018; 376(2):125-35.
6. Kendi A, Tuba TR, Halfdanarson AP, et al. “Therapy With 177Lu-DOTATATE: Clinical Implementation and Impact on Care of Patients With Neuroendocrine Tumors.” *Am J Roentg*, 2019; 213(2):309-17.
7. Rogoza O, Megnis K, Kudrjavceva M, et al. “Role of Somatostatin Signaling in Neuroendocrine Tumours.” *Int J Mol Sci*, 2022; 23(3):1447.
8. Rindi G, Wiedenmann G. “Neuroendocrine neoplasms of the gut and pancreas: new insights.” *Nature Reviews Endocrinology*, 2012; 8(1):54-64.
9. Hofman MS, Lau WFE, Hicks RJ. “Somatostatin Receptor Imaging with 68Ga DOTATATE PET/CT: Clinical Utility, Normal Patterns, Pearls, and Pitfalls in Interpretation.” *Radiographics*, 2015; 35(2).
10. Costanzi E., Simioni C., Conti I., Laface I., Varano G., Brenna C., Neri L.M. “Two Neuroendocrine G Protein-coupled Receptor Molecules, Somatostatin and Melatonin: Physiology of Signal Transduction and Therapeutic Perspectives.” *J Cell Physiol*, 2021;236:2505–2518.
11. Halfdanarson TR, Strosberg JR, Tang L, Bellizzi AM, Bergsland EK, O'Dorisio TM, Halperin DM, Fishbein L, Eads J, Hope TA, Singh S, Salem R, Metz DC, Naraev BG, Reidy-Lagunes DL, Howe JR, Pommier RF, Menda Y, Chan JA. “The North American Neuroendocrine Tumor Society Consensus Guidelines for Surveillance and Medical Management of Pancreatic Neuroendocrine Tumors.” *Pancreas*, 2020; 49(7):863-881.
12. de Keizer B, van Aken MO, Feelders RA, et al. “Hormonal crises following receptor radionuclide therapy with the radiolabeled somatostatin analogue [^177^Lu-DOTA0,Tyr3]octreotate.” *Eur J Nucl Med Mol Imaging,* 2008; 35:749–755.
13. Gade AK, Olariu E, Douthit NT. “Carcinoid Syndrome: A Review.” *Cureus*, 2020; 12(3):e7186.
14. Tapia Rico G, Li M, Pavlakis N, Cehic G, Price TJ. “Prevention and management of carcinoid crises in patients with high-risk neuroendocrine tumours undergoing peptide receptor radionuclide therapy (PRRT): literature review and case series from two Australian tertiary medical institutions.” *Cancer Treat Rev*, 2018; 66:1–6.
15. del Olmo-Garcia MI, Muros MA, Lopez-de-la-Torre M, et al. “Prevention and Management of Hormonal Crisis during Theragnosis with LU-DOTA-TATE in Neuroendocrine Tumors. A Systematic Review and Approach Proposal.” *J Clin Med*, 2020; 9(7).
16. Dhanani J, Pattison DA, Burge M, et al. “Octreotide for resuscitation of cardiac arrest due to carcinoid crisis precipitated by novel peptide receptor radionuclide therapy (PRRT): A case report.” *J Crit Care*, 2020; 60:318-322.
17. Baradasi C, Benatti S, Luppi G, Garajova I, Piacentini F, Dominici M, Gelsomino F. “Carcinoid Crisis: A Misunderstood and Unrecognized Oncological Emergency.” *Cancers*, 2022; 14(3):662.
18. Mittra E. “Neuroendocrine Tumor Therapy: 177Lu-DOTATATE.” *AJR Am J Roentgenol*, 2018; 211(2):278-85.
19. Cheng Y, Anthony L, Delcher C, Moga DC, Chauhan A, Huang B, Adams V. “Prescribing Characteristics of Octreotide Immediate-Release and Long-Acting Release in Patients with Neuroendocrine Tumors.” *Oncologist*, 2023; 28(6):479-85.
20. Burkett BJ, Dundar A, Young JR, et al. “How We Do It: A Multidisciplinary Approach to 177Lu DOTATATE Peptide Receptor Radionuclide Therapy.” *Radiol*, 2020; 298(2):261-274.
21. Hicks RJ, Kwekkeboom DJ, Krenning E, Bodei L, Grozinsky-Glasberg S, Arnold R, Borbath I, Cwikla J, Toumpanakis C, Kaltsas G, Davies P. “ENETS consensus guidelines for the standards of care in neuroendocrine neoplasms: peptide receptor radionuclide therapy with radiolabelled somatostatin analogues.” *Neuroendocrinology*, 2017; 105(3):295-309.
22. Rolleman EJ, Kooij PP, de Herder WW, Valkema R, Krenning, EP, de Jong M. “Somatostatin receptor subtype 2-mediated uptake of radiolabelled somatostatin analogues in the human kidney.” *Eur J Nuc Med Mol Imag*, 2007; 34:1854-60.

Statements and Declarations:

Funding: The authors declare that no funds, grants, or other support were received during the preparation of this manuscript.

Competing Interests: The authors have no relevant financial or non-financial interests to disclose.

Authorship: All authors contributed to the study conception and design. Material preparation, data collection and analysis were performed by Stephen Sozio, Murray Becker, and Jeffrey Kempf. The first draft of the manuscript was written by Stephen Sozio and all authors commented on previous versions of the manuscript. All authors read and approved the final manuscript.

Ethics Approval: This is a systematic review which does not include any identifiable protected health information. Thus, no ethical approval or consent is required.

Data Availability: Data sharing not applicable to this article as no datasets were generated or analyzed during the current study.
