# Supplementary material for: Carcinoid crisis in Lutetium-177-Dotatate therapy of neuroendocrine tumors: an overview of pathophysiology, risk factors, recognition, and treatment
Source: EJNMMI Rep. 2024 Sep 13;8(1):29. doi: 10.1186/s41824-024-00216-6 (PMC11393224; doi:10.1186/s41824-024-00216-6)
Supplement: Supplementary file 2 — Supplementary Material 2 [file 41824_2024_216_MOESM2_ESM.docx]

Reviewer #1: Thank you for this interesting manuscript on the pathophysiology, risk factors, recognition, and treatment of carcinoid crisis potentially induced by the lutetium-177-dotatate therapy.
Due to the growing interest in the use of radionuclide therapy in the management of NETs, it is indeed necessary to recognize and treat carcinoid syndrome early to reduce morbidity. In addition, the authors present their experience with lutetium-177-associated complications over a five-year period.
The review covers the main clinical considerations.  The topic is of interest.

Comments:

1. Title should be rephrased. The paper looks more like an overview related to carcinoid syndrome rather than a literature review.

The title has now been revised to “Carcinoid Crisis in Lutetium-177-Dotatate Therapy: An Overview of Pathophysiology, Risk Factors, Recognition, and Treatment.” Additionally, we have revised the manuscript in its entirety to characterize our purpose as providing an overview of the topic, as opposed to a comprehensive literature review.

2. Abstract: Please eliminate the sentence "Management of carcinoid…. reducing morbidity» from the results. It's more a conclusion which you have already included in the conclusions.

This sentence has been removed.

3. Methods: The authors should clarify how they made the review (articles from pubmed? others?), how many articles were included, the inclusion and exclusion criteria… if you talk about "A comprehensive literature review" that information should be included. Since various aspects were considered, perhaps the research was subdivided according to these aspects? Please comment.

We have further expanded upon our methods, as suggested.

4. Literature review: again, looks more like an overview of the NETs, carcinoid syndrome, treatment options, pathology, risk factors … rather than a literature review. In my opinion, one could divide this part into two chapters: an overview of NETs and pathophysiology and focus the review on the aspects that actually represent the topic of this manuscript, i.e. knowledge of risk factors, early recognition and treatment. Tip: insert tables with the various articles included in the review and their clinical relevance (according to topic), i.e. treatment management.

We have reframed the purpose of our manuscript as an overview to succinctly present clinicians, trainees, and other allied readers with key/salient information related to NETs and lutetium-177-dotatate therapy-induced carcinoid crisis, which can be easily and rapidly integrated into a provider’s clinical practice.

Of specific importance, we have revised the overview to be limited to salient information which enhances the understanding of lutetium-177-dotatate therapy-induced carcinoid crisis, as subsequently presented in the manuscript. Additionally, we have revised the structure and content of the manuscript body to better reflect a natural progression from background information to details of lutetium-177-dotatate therapy-induced carcinoid crisis, and included a table of each article’s relevance to the topic, as suggested.

5. Page 5, line 9: please eliminate "While Table 2 highlights the most common symptoms of carcinoid crisis", somehow repeats itself (see line 7). Please reformulate.

This sentence has been removed.

6. Page 5, line 44-46: please insert citation at the end: "Review of literature revealed…. octreotide infusion. "

A citation has been added.

7. Page 6, line 43-45: Patients treated at your institution with lutetium-177-Dotatate have not reported any complications of carcinoid crisis. It would be interesting if you could provide some clinical information about these patients, type of tumour, presence or absence of risk factors for carcinoid syndrome, etc. … and discuss the correlation with what is already known in the literature.

This feedback is in alignment with what our group had originally planned to include in this manuscript! We initially intended to include a comprehensive review of the demographics and characteristics of patients whom we have treated to date, with further details in those who experienced adverse event(s) related to therapy. Unfortunately, due to institution-related constraints, we were unable to provide additional details regarding patients who had not experienced an adverse event related to treatment. Thus, we were limited to providing details solely regarding the one patient who had experienced an adverse event related to therapy. However, our group is strongly considering a future manuscript detailing the characteristics and clinical course of those whom have undergone therapy (regardless of presence/absence of adverse event(s)), to include each person’s risk factors for carcinoid syndrome, and correlating our findings to current literature.

Thank you for your feedback!

Reviewer #2: The quality of the present review should be improved.
In details:

- The abstract refers to e literature review and an institutional review of the PRRT induced carcinoid crisis
- The literature review lacks considerable detail -the standard methodology for reporting a literature search, the details of the studies captured by the literature review and the selection criteria are lacking. Please add.

In alignment with the feedback provided by Reviewer #1, we have revised the title and body of the manuscript to better reflect its purpose of providing an overview of relevant literature (as opposed to a comprehensive literature review), as well as details regarding the how our overview was performed in the Methods section. The fundamental methodology of this overview is to succinctly present clinicians, trainees, and other allied readers with key/salient information related to the topic which could be easily and rapidly integrated into a provider’s clinical practice, with the additional unique value of a summary of our own institution’s experiences regarding incidences of carcinoid syndrome and adverse events in the setting of lutetium-177-dotatate therapy.

- The Literature review describes Neuroendocrine tumors in general and their management- this is not fully relevant in regards to the title of this paper. - much of this material should be in the Background.

We have revised the title of the manuscript to better reflect the purpose of our work to provide an overview of the topic. We ultimately included a background of neuroendocrine tumors most significantly to provide context as NETs are less commonly encountered than other neoplasms, we felt a brief review of their characteristics and would be beneficial to the reader:

- One sentence detailing the most common NET locations is employed in this section, which is an important component of risk factors of developing carcinoid syndrome in lutetium-177-dotatate therapy, as subsequently discussed
- Pathophysiology of the hormones that NETs release, and a brief overview of the cellular mechanisms in which these hormones are released, serves a fundamental building block for presenting the pathophysiology of carcinoid syndrome, and subsequently how different treatment(s) function to achieve desired effect
- As nuclear imaging is commonly performed in the evaluation of suspected or known NETs, we expanded upon the previously presented molecular mechanisms to briefly explain the mechanism and role of imaging in the setting of NETs, which is required to be performed prior to undergoing treatment. To better promote a logical flow of the manuscript, this paragraph has been relocated from its original position
- A brief overview of the approach to treatment of NETs provides contextual evidence for how lutetium-177-dotatate therapy is broadly employed in the treatment plan of NETs

- The results of the literature review should be better summarized - a Table summary of the reported papers in regard to the paper's topic would be essential.

We have revised the manuscript to include a table of each article’s relevance to the topic.

- A systematic review would be useful here to deduce the relative importance of the clinical factors for patients at risk of a crisis.

The literature specific to the relative importance of risk factors for developing carcinois crisis is comparatively sparse, with data regarding the integration and translation of such information into the culmination of a risk:benefit decision regarding proceeding with treatment and/or preventive pre-treatment measures to be highly specific and nuanced to each patient. Thus, we found the utility of providing such analysis in this specific manuscript would be limited. Further, we found completing a systematic review of such would result in an excessively bulky sub-section which would detract from the other salient details surrounding lutetium-177-dotatate carcinoid crisis. In alignment with our goal of providing a high-level summary of the key/salient information related to lutetium-177-dotatate therapy and carcinoid crisis which could be easily and rapidly integrated into a provider’s clinical practice, our group elected to include an easy-to-read table of the most common clinical factors imparting risk of developing therapy-related carcinoid crisis.

However, given the importance of this topic and potential importance of such information in advancing knowledge of how to approach lutetium-177-dotatate-induced carcinoid crisis, our group is actively in discussion for completing this suggested formal systematic review of these risk factors, with the ultimate goal of ranking their relative importance based on such review, for a separate manuscript in the near-future.

- The institutional review is lacking in significant detail regarding patient demographics, etc. The case that is presented is not relevant here.

We initially intended to include a comprehensive review of the demographics and characteristics of patients whom we have treated to date, with further details in those who experienced adverse event(s) related to therapy. Unfortunately, due to institution-related constraints, we were unable to provide additional details regarding patients who had not experienced an adverse event related to treatment. Thus, we were limited to providing details solely regarding the one patient who had experienced an adverse event related to therapy. While we understand the presented case is not one of carcinoid syndrome, this was the sole incidence of an adverse event related to therapy that we noted after over a half decade of administering therapies. Furthermore, as our literature review revealed no detailed documentation of such adverse effect specific to the setting of lutetium-177-dotatate therapy, we elected to include this event and its details in the manuscript for further added value of this work.

Our group is strongly considering a future manuscript detailing the characteristics and clinical course of those whom have undergone therapy (regardless of presence/absence of adverse event(s)), to include each person’s risk factors for carcinoid syndrome, and correlating our findings to current literature.

Thank you for your feedback!
